# Cyclooxygenase-2 Inhibition Limits Angiotensin II-Induced DNA Oxidation and Protein Nitration in Humans

**DOI:** 10.3389/fphys.2017.00138

**Published:** 2017-03-10

**Authors:** Vincent Pialoux, Marc J. Poulin, Brenda R. Hemmelgarn, Daniel A. Muruve, Erica N. Chirico, Camille Faes, Darlene Y. Sola, Sofia B. Ahmed

**Affiliations:** ^1^Laboratoire Interuniversitaire de Biologie de la Motricité EA7424, Université de Lyon, Université Claude Bernard Lyon 1Villeurbanne, France; ^2^Faculty of Medicine, Hotchkiss Brain Institute, University of CalgaryCalgary, AB, Canada; ^3^Department of Physiology and Pharmacology, Faculty of Medicine, University of CalgaryCalgary, AB, Canada; ^4^Faculty of Medicine, Libin Cardiovascular Institute of Alberta, University of CalgaryCalgary, AB, Canada; ^5^Department of Clinical Neurosciences, Cumming School of Medicine, University of CalgaryCalgary, AB, Canada; ^6^Faculty of Kinesiology, University of CalgaryCalgary, AB, Canada; ^7^Department of Medicine, Faculty of Medicine, University of CalgaryCalgary, AB, Canada; ^8^Department of Biomedical Sciences, Cooper Medical School of Rowan UniversityCamden, NJ, USA

**Keywords:** blood pressure, celecoxib, cycloxygenase-2 inhibition, endothelin-1, humans, oxidative stress, nitric oxide, renin-angiotensin system

## Abstract

Compared to other cyclooxygenase-2 inhibitors, celecoxib is associated with a lower cardiovascular risk, though the mechanism remains unclear. Angiotensin II is an important mediator of oxidative stress in the pathophysiology of vascular disease. Cyclooxygenase-2 may modify the effects of angiotensin II though this has never been studied in humans. The purpose of the study was to test the effects of selective cyclooxygenase-2 inhibition on plasma measures of oxidative stress, the vasoconstrictor endothelin-1, and nitric oxide metabolites, both at baseline and in respose to Angiotensin II challenge in healthy humans. Measures of 8-hydroxydeoxyguanosine, advanced oxidation protein products, nitrotyrosine, endothelin-1, and nitric oxide metabolites were assessed from plasma samples drawn at baseline and in response to graded angiotensin II infusion (3 ng/kg/min × 30 min, 6 ng/kg/min × 30 min) before and after 14 days of cyclooxygenase-2 inhibition in 14 healthy subjects (eight male, six female) in high salt balance, a state of maximal renin angiotensin system suppression. Angiotensin II infusion significantly increased plasma oxidative stress compared to baseline (8-hydroxydeoxyguanosine; +17%; advanced oxidation protein products; +16%), nitrotyrosine (+76%). Furthermore, levels of endothelin-1 levels were significantly increased (+115%) and nitric oxide metabolites were significantly decreased (−20%). Cycloxygenase-2 inhibition significantly limited the increase in 8-hydroxydeoxyguanosine, nitrotyrosine and the decrease in nitric oxide metabolites induced by angiotensin II infusion, though no changes in advanced oxidation protein products and endothelin-1 concentrations were observed. Cyclooxygenase-2 inhibition with celecoxib partially limited the angiotensin II-mediated increases in markers of oxidative stress in humans, offering a potential physiological pathway for the improved cardiovascular risk profile of this drug.

## Introduction

Compared to other cyclooxygenase-2 inhibitors, celecoxib is associated with a lower cardiovascular risk (Solomon et al., 2004; Hirayama et al., 2014; Nissen et al., 2016; Gunter et al., 2017). Though the mechanism is unclear, animal studies suggest that COX-2-inhibition plays a role in attenuating the effects of Angiotensin II (AngII; Wu et al., 2005; Martínez-Revelles et al., 2013) the effector molecule of the renin angiotensin system (RAS), which plays a central role in the pathophysiology of hypertension, an important risk factor for cardiovascular disease (Touyz and Schiffrin, 2004; Mehta and Griendling, 2007). The actions of AngII result in not only vasoconstriction but also generation of reactive oxygen species (ROS; Mehta and Griendling, 2007) stimulation of endothelin-1 (ET-1; Sasser et al., 2002; Montanari et al., 2003) and depletion of nitric oxide (NO) vascular bioavailability (Thomas et al., 2008) all of which have been shown to promote atherosclerosis and increase vascular risk (Touyz and Schiffrin, 2004), though in some circumstances, ROS activation may be beneficial (Le Moal et al., 2017). Interestingly, ET-1 synthesis and NO metabolism inhibition, both of which induce vasoconstriction, have been shown to be ROS-mediated (Sedeek et al., 2003; Thomas et al., 2008). It is therefore possible that increased ROS production acts as a mechanism for AngII to increase the vasoconstrictor/vasodilator ratio. Previous human studies have shown a role for endothelin-1 through measurement of hemodynamics after endothelin-A blockade (Montanari et al., 2003) but we are not aware of any studies that have simultaneously examined changes in oxidative stress, endothelin-1 levels and nitric oxide metabolites in response to an AngII challenge. Moreover, animal studies suggest that cyclooxygenase-2 plays a role in attenuating these effects, mainly via the reduction of the NADPH oxidase-dependent superoxide anion generation (Martínez-Revelles et al., 2013; Wu et al., 2005), suggesting that inhibition of this enzyme could blunt the unfavorable oxidative stress effects of AngII, though this has never been studied in humans.

Thus, the purpose of this study was two-fold: to determine to effects of selective cyclooxygenase-2 inhibition on plasma measures of oxidative stress, the vasoconstrictor endothelin-1 and nitric oxide metabolites, both at baseline and in response to Angiotensin II challenge in healthy humans. We hypothesized that selective COX-2 inhibition would reduce markers of oxidative and nitrosative stress, both at baseline and in response to angiotensin II challenge.

## Materials and methods

### Ethics approval

The study protocol was approved by the Conjoint Health Research Ethics Board at the University of Calgary and conformed to the standards set by the latest revision of the Declaration of Helsinki. All subjects gave written informed consent.

### Subjects

Healthy, non-smoking, normotensive, non-diabetic, non-pregnant adult subjects ingesting no medications from the community were recruited to participate in the study. Subjects completed an initial medical history, physical examination, electrocardiogram, and laboratory screening. No subject was ingesting any medication; specifically, no women were ingesting the oral contraceptive as exogenous estrogen is known to affect RAS (Ahmed et al., 2004) and ROS activity (Finco et al., 2011).

### Protocol

Subjects consumed >200 mmol sodium/day for 3 days before the study. Sodium and creatinine excretion were measured from a 24 h urine collection or estimated from the second morning void (Kawasaki et al., 1993). Given that variations in menstrual cycle are associated with alterations in RAS (Chidambaram et al., 2002) and ROS (Cornelli et al., 2013), all female subjects were studied 14 days after the start of last menstrual period. Subjects were studied in the supine position after an 8 h fast. At 08:00 h, an intravenous catheter was placed in each arm (for infusion and blood sampling). Blood pressure (BP) was recorded every 15 min by an automatic recording device (Dinamap; Critikon, Tampa, FL). After a 90 min equilibration period to establish baseline hemodynamic measurements, a graded infusion of AngII at 3 ng/kg/min × 30 min (30 min), followed by 6 ng/kg/min × 30 min (60 min) was administered, followed by a 30 min recovery period (90 min) at which point the study ended. Plasma levels of oxidative stress (i.e., 8-hydroxy-2′-deoxyguanosine [8-OHdG]), advanced oxidation protein products [AOPP], total antioxidant status (ferric reducing antioxidant power, FRAP), nitrative stress (nitrotyrosine [3-N]) ET-1, and end-products of nitric oxide metabolism (NOx) as well as circulating components of the RAS (plasma renin activity, aldosterone), were collected at baseline and every 30 min until the end of the study (30, 60, and 90 min). Blood was collected in 7 ml ethylenediaminetetraacetic acid (EDTA) tubes. The plasma and serum were obtained by centrifugation of the samples at 1,000 g for 10 min at 4°C immediately and 20 min respectively after blood collection. Plasma and serum were separated into aliquots and frozen at −80°C until assays could be performed. Subjects then ingested the selective cyclooxygenase-2 inhibitor, celecoxib, at the clinical dose of 200 mg daily × 14 days, after which time they repeated the AngII challenge study day. Compliance with the medication was ensured by pill counting and regular telephone followup by the study nurse. Study subjects were also asked to return pill containers and any unused tablets.

### Biochemical analyses

Concentrations of plasma 8-OHdG were determined using an enzyme-linked immunosorbent assay (ELISA) kit from Cell BioLabs (Cell Biolabs, Inc., San Diego, CA), with limits of detection 1–200 μg.l^−1^. Since DNA is known to be very sensitive to ROS (Loft et al., 2008a), 8-OHdG, an end-product of DNA oxidation, is one of the most reliable markers for oxidative stress (Loft et al., 2008b) and was selected as the primary marker of oxidative stress in this study. Concentrations of plasma AOPP were determined using the semi-automated method described by Witko-Sarsat et al. (1996) and concentrations were expressed as micromoles per liter of chloramine-T equivalents. AOPP is one of the most reliable markers for oxidative stress in many inflammation processes (Witko-Sarsat et al., 1996; Pialoux et al., 2009b). Concentrations of plasma nitrotyrosine, an end-product of protein nitrosilation, were measured using an ELISA kit from Cell BioLabs (Cell Biolabs, Inc., San Diego, CA) with limits of detection 1–8000 nmol.l^−1^. Since tyrosine is sensitive to nitrosilation by peroxynitite (ONOO^−^), the amount of nitrotyrosine reflects the activity of ONOO^−^. Peroxynitite is formed by the reaction of the superoxide anion (${\text{O}}_{2}^{.-}$) with NO. Plasma ferric reducing antioxidant power (FRAP) was assessed according to the manual Benzie and Strain (Benzie and Strain, 1996) method and were measured by spectrophotometry at 37°C. FRAP concentration was calculated using an aqueous solution of known Fe^2+^ concentration (FeSO_4_, 7H_2_O_2_) as standard at a wavelength of 593 nm. FRAP is a global indicator of antioxidant power. It has been reported that FRAP is negatively correlated with oxidative stress after hypoxic exposure (Pialoux et al., 2009a), highlighting the potential sensitivity of FRAP to oxidative stress. Concentrations of plasma endothlin-1 were measured using an ELISA kit (Cell Biolabs Inc., San Diego, CA). The end-products of nitric oxide (NOx), nitrites, and nitrates, were measured using a commercially available colorimetric kit (Cayman Chemical Company, Ann Arbor, MI, USA). A radioimmunoassay (RIA) was utilized for plasmas renin activity (PRA; DiaSorin Clinical Assays, Stillwater, MN, USA). In brief, Angiotensin I (AngI), the primary product of PRA was generated at 37°C from endogenous renin and renin substrate at pH 6.0. The integrity of the generated AngI was maintained by inhibition of proteolytic activity using EDTA and phenylmethylsufonyl fluoride in the generation system. The accumulated AngI reflects PRA under these controlled conditions. The AngI generated was determined by RIA using competitive binding principles, where the antibody was immobilized onto the lower inner wall of coated tubes. Aldosterone was also measured using an RIA assay. AngII plasma levels were measured by standard laboratory immunoassay techniques (Quest Diagnostics; San Juan Capistrano, CA, USA). Glycated hemoglobin was measured using a colorimetric method (Integra 800 CTS, Roche, USA). Plasma triglycerides were measured by enzymatic colorimetric assay (Cobas 6,000, Roche, USA). Cholesterol was measured by enzymatic colorimetric assay (Cobas 8,000, Roche, USA).

### Statistical analyses

Data are reported as mean ± standard deviations (*SD*) unless otherwise indicated. The primary analysis of this exploratory study tested the change in measures of oxidative stress, ET-1, and NO at baseline and in response to a graded AngII challenge, before and after COX-2 inhibition using a two-way repeated measure ANOVA followed by Sidak *post-hoc* test. Statistical analyses were performed using Stata (version 10.0; Stata, College Station, TX) with two-tailed significance levels of 0.05.

## Results

### Baseline characteristics

Characteristics of the 14 study subjects are presented in Table 1. Subjects were normotensive, non-obese, non-diabetic, and in high-salt balance, a state of maximal RAS suppression, as indicated by urine sodium excretion.

**Table 1:** **Baseline and anthropometric characteristic of the subjects**.

	**Males (*n* = 8)**	**Females (*n* = 6)**
Age (years)	32.3 ± 7.9	37.5 ± 15.4
Weight (kg)	84.4 ± 17.4	62.4 ± 8.6
Height (cm)	180.3 ± 11.3	162.7 ± 4.9
BMI (kg/m^2^)	25.6 ± 3.7	24.0 ± 3.7
Fat mass (%)	19.7 ± 4.7	29.1 ± 7.1
Glycaemia (mmol/L)	4.90 ± 0.35	5.00 ± 0.44
HbA1c (%)	5.22 ± 0.20	5.48 ± 0.20
Cholesterol (mmol/L)	4.12 ± 0.71	4.32 ± 0.65
Triglycerides (mmol/L)	1.22 ± 0.56	0.77 ± 0.41
24 h Na excretion (mmol/day)	405 ± 102	355 ± 73
24 h Cr excretion (mg/day)	2305 ± 334	1260 ± 160

### Responses to AngII challenge Pre-COX-2 inhibition

**Figure 1:**
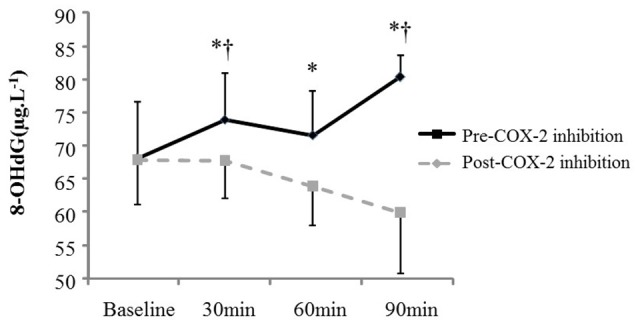
**Plasma oxidative stress (8-OHdG) at baseline and in response to Angiotensin II infusion, pre- and post COX-2 inhibition**. ^*^p < 0.05 compared to corresponding baseline value. ^†^*p* < 0.05 vs. corresponding timepoint pre-COX-2 inhibition.

**Figure 2:**
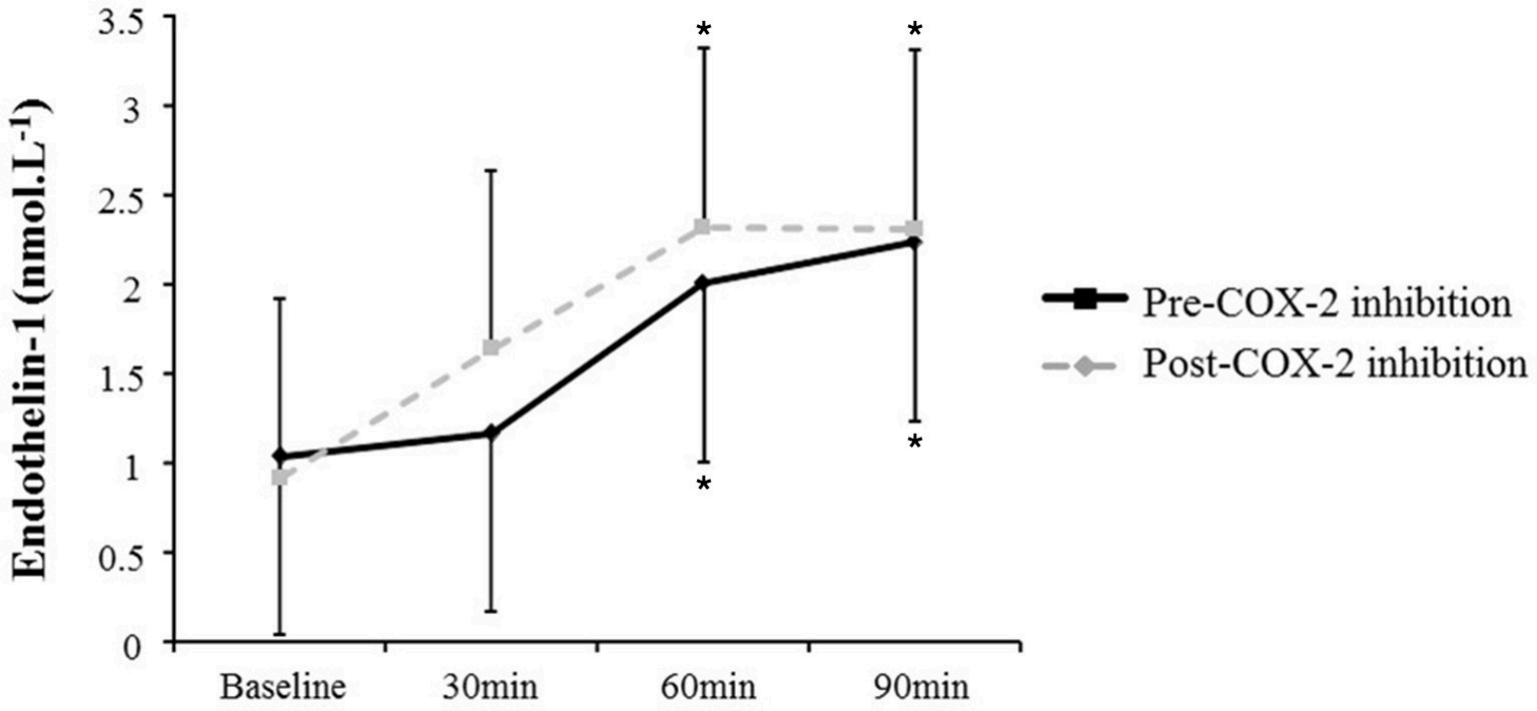
**Plasma endothelin-1 at baseline and in response to Angiotensin II infusion, pre- and post-COX-2 inhibition**. ^*^p < 0.05 compared to corresponding baseline value.

AngII increased all measures of oxidative stress (Table 2; 8-OHdG: +17%, *p* = 0.02 at 90 min (Figure 1); AOPP: +15 and +16%, *p* = 0.01 at 30 and 60 min), protein nitration (+76%, *p* = 0.004 at 60 min), FRAP: −13 and −14%, *p* = 0.04 at 60 and 90 min). Similarly, AngII challenge resulted in an increase in ET-1 (+93 and +115%, *p* < 0.001 at 60 min and 90 min; Figure 2) and a decrease in NOx (−20%, *p* = 0.02 at 60 and 90 min).

**Table 2:** **Measures of plasma oxidative stress, endothelin-1, products of nitric oxide metabolism, aldosterone and plasma renin activity at baseline and in response to Angiotensin II infusion, pre- and post-cyclooxygenase-2 inhibition**.

	**Pre-COX-2 inhibition**				**Post-COX-2 inhibition**			
	**Baseline**	**30 min**	**60 min**	**90 min**	**Baseline**	**30 min**	**60 min**	**90 min**
AOPP (μmol.L^−1^)	83.2 ± 18.0	95.5 ± 22.6^*^	96.3 ± 21.8^*^	89.2 ± 22.8	69.5 ± 17.9	103.3 ± 24.6^*^	94.3 ± 23.4^*^	97.1 ± 22.5^*^
3-N (nmol.L^−1^)	23.5 ± 9.6	24.8 ± 10.1	41.4 ± 10.2^*^	24.0 ± 11.0	23.0 ± 9.6	27.7 ± 11.0	29.0 ± 13.1^†^	29.4 ± 17.8
FRAP (mmol.L^−1^)	1116 ± 107	1082 ± 151	975 ± 55^*^	957 ± 142^*^	996 ± 169	1104 ± 203	904 ± 94	877 ± 67
ET-1 (nmol.L^−1^)	1.04 ± 0.45	1.17 ± 0.36	2.01 ± 0.64^*^	2.24 ± 0.54^*^	0.92 ± 0.40	1.64 ± 1.28	2.32 ± 2.26^*^	2.31 ± 1.41^*^
NOx (μmol.L^−1^)	31.0 ± 5.7	27.4 ± 3.2	24.9 ± 3.0^*^	24.9 ± 4.1^*^	27.2 ± 7.1	26.1 ± 5.7	28.1 ± 5.5	25.8 ± 7.1
PRA (ng.mL^−1^.h^−1^)	0.26 ± 0.14	0.14 ± 0.09^*^	0.10 ± 0.07^*^	0.10 ± 0.07^*^	0.19 ± 0.15	0.13 ± 0.11^*^	0.11 ± 0.10^*^	0.12 ± 0.11^*^
Aldosterone (pmol.L^−1^)	175 ± 88	225 ± 134	357 ± 184^*^	282 ± 123^*^	125 ± 68^†^	190 ± 118	291 ± 158^*^	240 ± 129^*^

*Values are presented as means ± SD. AOPP, advanced oxidation protein products; 3-N, nitrotyrosine; FRAP, ferric reducing antioxidant power; ET-1, Endothelin-1; NOx, end-products of nitric oxide metabolism; PRA: Plasma renin activity*.

* *p < 0.05 vs. corresponding baseline*;

† *p < 0.05 vs. pre-COX-2 inhibition at same time point*.

As anticipated, all subjects demonstrated significant changes in all indices of blood pressure and circulating RAS components in response to AngII challenge compared with baseline values (Tables 2, 3).

**Table 3:** **Measures hemodynamic parameters at baseline and in response to Angiotensin II infusion, pre- and post-cyclooxygenase-2 inhibition**.

	**Pre-COX-2 inhibition**				**Post-COX-2 inhibition**			
	**Baseline**	**30 min**	**60 min**	**90 min**	**Baseline**	**30 min**	**60 min**	**90 min**
SBP (mmHg)	117 ± 15	129 ± 18^*^	131 ± 15^*^	118 ± 14	115 ± 12	127 ± 12^*^	130 ± 13^*^	114 ± 11
DBP (mmHg)	70 ± 9	81 ± 9^*^	80 ± 11^*^	70 ± 8	68 ± 8	80 ± 10^*^	81 ± 11^*^	68 ± 10
MAP (mmHg)	86 ± 10	97 ± 11^*^	97 ± 12^*^	86 ± 9	84 ± 9	96 ± 10^*^	98 ± 11^*^	84 ± 9

*Values are presented as means ± SD. SBP, systolic blood pressure; DBP, diastolic blood pressure. MAP, mean arterial pressure; PRA, Plasma renin activity*.

* *p < 0.05 vs. corresponding baseline*.

### Measures of oxidative stress, ET-1, and NOx in responses post-COX-2 inhibition

The changes in measures of oxidative stress, ET-1, and NOx after COX-2 inhibition are outlined in Table 2 and Figures 1, 2. COX-2 inhibition abolished the increase in both 8-OHdG and nitrotyrosine and the decrease in FRAP and NOx observed in response to AngII challenge pre-COX-inhibition, though no differences in the AOPP and ET-1 responses to AngII infusion were observed. No difference was observed in the hemodynamic and plasma renin activity responses to COX-2 inhibition (Tables 2, 3). However, COX-2 inhibition significantly decreased baseline aldosterone levels (*p* = 0.03) and blunted the aldosterone increase in response to AngII (*p* = 0.01; all *p*-values vs. pre-COX-2 inhibition at same time point).

## Discussion

To our knowledge, this is the first study to examine the effects of COX-2 inhibition on AngII-mediated increases of oxidative stress, in addition to ET-1, a potent vasoconstrictor, and NO metabolites, a proposed biological marker of vasodilatation capacities and endothelial function (Heiss et al., 2006) in healthy humans. Our key findings were as follows: (1) AngII induced an increase in markers of oxidative stress; (2) AngII upregulated ET-1; (3) AngII decreased measures of NO metabolites; and (4) inhibition of COX-2 limited the AngII-mediated increase in markers of oxidative stress. Our results expand on previous animal studies (Wu et al., 2005; Martínez-Revelles et al., 2013), and suggest that in addition to vasoconstriction, acute exposure to AngII results in an unfavorable oxidative stress profile with an increased vasoconstrictor agent (endothelin-1) to vasodilator ratio offering a potential pathophysiological mechanism for the endothelial dysfunction observed in high cardiovascular risk populations. In this context, celecoxib appears to favorably modulate the increase in oxidative stress in the setting of acute AngII exposure.

### Responses to AngII challenge

Previous studies have suggested a role for AngII in the generation of ROS (Sifi et al., 2017) More specifically, AngII modulates vasoconstriction by acting through the AngII type 1 receptors located in vascular smooth muscle (Mehta and Griendling, 2007) and growing evidence suggests that such detrimental effects of AngII type 1 receptor activation are mediated by oxidative stress through the activation of nicotinamide adenine dinucleotide phosphate (NADPH) oxidase (Mehta and Griendling, 2007; Li et al., 2016). The increase in blood pressure resulting from AngII infusion may also in turn augments ROS production via an increased shear stress. AngII induces a transient increase in vascular smooth muscle cell COX-2 mRNA accumulation and COX-2-derived prostanoid may contribute to the pathophysiological conditions associated with elevated levels of Ang II in the vasculature (Young et al., 2000).

Because endothelin-1 is known to be a potent vasoconstrictor and mitogen it likely plays a role in the development of hypertension (Channick et al., 2001; Remuzzi et al., 2002; Lin et al., 2015). In our study, endothelin-1 was clearly upregulated by AngII infusion. This result is consistent with other studies showing that AngII stimulates the production and release of endothelin-1 (Pollock, 2005). Although controversial, some authors suggest that hypertensive effects of AngII could be mediated in part by endothelin-1 (Rossi et al., 1999). The hypothesis that AngII stimulates the production and release of endothelin-1 is supported by *in vitro* (Moreau et al., 1997; Hong et al., 2004) and human (Jilma et al., 1997) studies. In rats, endothelin-1 augmented the pressor response to angiotensin II infusion (Yoshida et al., 1992). Interestingly, recent *in vivo* rat data suggest that endothelin-1-induced vasoconstriction may be dependent on the production of ROS (Thomas et al., 2008). It is therefore possible that increased production of ROS could be a mechanism for AngII to induce endothelin-1 synthesis as occurs in the vascular dysfunction observed in the setting of chronic kidney disease (Wang et al., 2015). Conversely, the bioavailability of the potent vasodilator NO has been proposed to be highly dependent on redox status. Under pathophysiological conditions, an increase in ROS through activation of AngII has been shown to decrease NO bioavailability (Sedeek et al., 2003). More specifically, ROS inactivates NO synthesized by endothelial nitric oxide synthase (eNOS) and can induce an uncoupling of eNOS (Sedeek et al., 2003).

### Responses to COX-2 inhibition

This study is the first to report in humans that the COX-2 inhibitor celocoxib attenuates AngII-induced oxidative stress and decrease in NO metabolites. It has been previously shown that selective COX-2 inhibition by celecoxib reduces oxidized LDL in patients with coronary artery disease (Chenevard et al., 2003). It has been demonstrated that COX-2 plays a role in attenuating the AngII pro-oxidant effects via the reduction of the NADPH oxidase-dependent superoxide anion generation (Wu et al., 2005). Similar results were observed using a genetic approach: COX-2 knockout mice demonstrate blunted Ang II-induced oxidative stress and increase in blood pressure (Wu et al., 2011). Taken together, these data suggest that celecoxib has antioxidant properties and may lead to the preservation of NO bioavailability and reduction of 3-NT as a stimulant of peroxynitrite activity under AngII challenge (Senbel et al., 2014).

Nevertheless, in our study, the lack of change in measures of oxidative stress at baseline highlight that the potential COX-2 inhibition associated-antioxidant properties appear to manifest only in the setting of an acute AngII challenge. In support of this, it has been demonstrated that angiotensin type 1 receptor (AT1R) blockers inhibit oxidative stress activity in the heart in the setting of hypertension (Bayorh et al., 2003; Dohi et al., 2003) independently of their effect on blood pressure.

Although celecoxib globally decreased aldosterone, restored NOx and potentially NO metabolism in response to AngII infusion, no changes in blood pressure either at rest or in response to AngII infusion were observed, son. These data in humans confirm previous studies demonstrating that chronic celecoxib treatment did not modify blood pressure in 2 kidney-1 clip hypertension model rats (Richter et al., 2004). The efficiency of selective COX-2 inhibition on systolic blood pressure may be related to the model of hypertension-induced (deoxycorticosterone acetate-salt model *vs*. our AngII infusion model; Okumura et al., 2002).

In rats with induced cardiac hypertrophy, treatment with celecoxib for 2–4 weeks suppressed pressure overload induced cardiac hypertrophy via decreasing apoptosis, inflammation, and oxidative stress (Zhang et al., 2016). In contrast and highlighting a potential difference between the effects of selective COX-2 inhibitors, rats who underwent chronic treatment with rofecoxib decreased oxidative stress through the reduction of the NADPH oxidase-dependent superoxide anion generation (Wu et al., 2005). Interestingly, this study reported that rofecoxib also blunted the hypertensive response to 12 days of AngII treatment. The differences between the reported effect of COX-2 inhibition on blood pressure in this study and our results may reflect both medication and interspecies differences as well as distinctions between acute vs. chronic AngII exposure. Indeed, in healthy rats, the superoxide dismutase mimetic tempol did not have any effect on the pressor or renal hemodynamic response to acute AngII infusion although it is able to reduce increases in mitogen activated protein kinase activation and decreases in glomerular filtration rate (López et al., 2003; Zhang et al., 2004). Finally, Baber et al. (2005) showed that systemic pressor responses to AngII infusion were not modified by COX-1 or COX-2 inhibitors whereas it was attenuated by losartan an AT1R antagonist.

Despite the beneficial effects of NO and aldosterone responses to AngII associated with exposure to celecoxib, no differences in the endothelin-1 response to AngII challenge were observed. This lack of effect in endothelin-1 may also explain the fact lack of change in the blood pressure response to AngII were observed with the ingestion of celecoxib despite the observed decreases in the NOx and aldosterone responses to AngII. Although endothelin-1 has been shown to play a role in modulating COX-2 and COX-2-derived prostaglandin E_2_ cellular expression (Spinella et al., 2004), it has not been shown to have a modulating effect of COX-2 on endothelin-1, consistent with our plasma endothelin 1 results.

The lack of change in the endothelin-1 response to AngII in the present study may represent the difference between acute and chronic exposures to AngII as the role of endothelin-1 in inducing the increase in superoxide was not observed during the first transient (measured after 10 min in this study) superoxide release induced by activation of the RAS (Laplante et al., 2005). This latter result could explain the divergent effect of celecoxib on oxidative stress and endothelin-1.

### Strengths and limitations

Healthy subjects were studied in the present investigation, and as such, the results may not be generalizable to patient populations. However, by including only healthy subjects, we were able to eliminate potential confounding factors such as diabetes, hypercholesterolemia, atherosclerosis, and obesity, which may have affected our primary outcome. In addition, although our findings are in keeping with animal studies using an acute AngII challenge (Wu et al., 2005; Martínez-Revelles et al., 2013), the shorter duration of AngII exposure may not mimic a chronically activated RAS and our results should be interpreted in this context. All the biomarkers of oxidative stress measured in this study have been shown to be associated with severity of cardiovascular disease (Ho et al., 2013). For example, the JUPITER trial supported the clinical utility of assessing inflammatory status in guiding intervention to limit cardiovascular events (Ridker et al., 2008). We assumed the change in circulating biomarkers in response to angiotensin II was reflective of vascular wall activity for the purposes of this study due to the necessary ethical limitations of human investigation. Interestingly, despite this potential limitation, our assays were sufficiently sensitive to detect the effect of Ang II infusion and celecoxib treatment as demonstrated by the significant difference between the conditions. The increase in blood pressure in response to Ang II may in turn augment ROS production via increased shear stress (Lo et al., 2013; Ding et al., 2015). However, since the Ang II infusion identical before and after celecoxib ingestion, the effects of COX-2 in inhibition on the oxidative stress response to Ang II infusion would not have been impacted. While sex-based differences in the vascular response to AngII challenge have been previously reported (Miller et al., 1999), this study was not powered to detect sex differences in outcomes. However, we established a priori that an individual subject's response to each intervention would be compared to his or her own baseline using a paired analysis, thus taking into account the sex of the subject. Furthermore, no differences were observed between the female and male response to cyclooxygenase-2 inhibition or angiotensin II infusion in this exploratory study. Finally, menstrual cycle variation and/or oral contraceptive use may affect ROS generation (Finco et al., 2011; Cornelli et al., 2013), but female subjects were studied at the mid-point of the menstrual cycle and women using exogenous sex hormones were excluded. Of the selective COX-2 inhibitors, only celecoxib is approved for sale in Canada; as such, we were not able to compare the effects of celecoxib to that of other selective COX-2 inhibitors. Our results demonstrate an association between celecoxib and blunting of the oxidative stress generated by angiotensin II in healthy humans independent of sex, though whether these findings apply to other selective COX-2 inhibitors is unknown.

## Conclusion

In summary, we have demonstrated that COX-2 inhibition with celecoxib limited the increase in markers of oxidative stress and the decrease in NO metabolites in response to AngII. Large, prospective studies are warranted to determine how the results of this study translate into clinical outcomes.

## Author contributions

VP and SA designed the study; VP, MP, BH, DM, EC, DS, and SA analyzed the data; VP, MP, BH, DM, EC, CF, DS, and SA interpreted the data; VP and SA drafted the manuscript; VP, MP, BH, DM, EC, CF, DS, and SA revised the manuscript critically for important intellectual content; VP, MP, BH, DM, EC, CF, DS, and SBA approved the final version of the manuscript submitted.

## Funding

VP is supported by the Institut Universitaire de France. Postdoctoral funding to VP (Supervisor MP) was provided by Alberta Innovates—Health Solutions (AIHS) and a Canadian Institutes for Health Research operating grant (PI MP). MP holds the Brenda Strafford Foundation Chair for Alzheimer Research. SA is an AIHS Clinical Investigator. This research was funded by an Alberta Heritage Foundation for Medical Research Establishment Grant to SA.

### Conflict of interest statement

The authors declare that the research was conducted in the absence of any commercial or financial relationships that could be construed as a potential conflict of interest.

## Abbreviations

3-N: nitrotyrosine
8-OHdG: 8-hydroxy-2'-deoxyguanosine
AngI: Angiotensin I
AngII: Angiotensin II
AOPP: advanced oxidation protein products
BP: blood pressure
COX-2: cyclooxygenase-2
EDTA: ethylenediaminetetraacetic acid
ELISA: enzyme-linked immunosorbent assay
ET-1: endothelin-1
FRAP: ferric reducing antioxidant power
NADPH: nicotinamide adenine dinucleotide phosphate
NO: nitric oxide
NOx: end-products of nitric oxide metabolism
${\text{O}}_{2}^{.-}$: superoxide anion
ONOO^−^: peroxynitite
PRA: plasma renin activity
RAS: renin angiotensin system
RIA: radioimmunoassay
ROS: reactive oxygen species.
